# The Human Cell Surfaceome of Breast Tumors

**DOI:** 10.1155/2013/976816

**Published:** 2013-09-09

**Authors:** Júlia Pinheiro Chagas da Cunha, Pedro Alexandre Favoretto Galante, Jorge Estefano Santana de Souza, Martin Pieprzyk, Dirce Maria Carraro, Lloyd J. Old, Anamaria Aranha Camargo, Sandro José de Souza

**Affiliations:** ^1^Ludwig Institute for Cancer Research, São Paulo 01308-050, SP, Brazil; ^2^Center for Applied Toxinology, Butantan Institute, São Paulo 05503-900, SP, Brazil; ^3^Centro de Oncologia Molecular Hospital Sírio-Libanês, São Paulo 01308-050, SP, Brazil; ^4^Instituto de Bioinformática e Biotecnologia, Natal 59064-560, RN, Brazil; ^5^Fluidigm Inc., South San Francisco 94080, CA, USA; ^6^Hospital AC Camargo, São Paulo 01509, SP, Brazil; ^7^Ludwig Institute for Cancer Research, New York 10017, NY, USA; ^8^Brain Institute, Federal University of Rio Grande do Norte, Natal 59064-560, RN, Brazil

## Abstract

*Introduction*. Cell surface proteins are ideal targets for cancer therapy and diagnosis. We have identified a set of more than 3700 genes that code for transmembrane proteins believed to be at human cell surface. *Methods*. We used a high-throuput qPCR system for the analysis of 573 cell surface protein-coding genes in 12 primary breast tumors, 8 breast cell lines, and 21 normal human tissues including breast. To better understand the role of these genes in breast tumors, we used a series of bioinformatics strategies to integrates different type, of the datasets, such as KEGG, protein-protein interaction databases, ONCOMINE, and data from, literature. *Results*. We found that at least 77 genes are overexpressed in breast primary tumors while at least 2 of them have also a restricted expression pattern in normal tissues. We found common signaling pathways that may be regulated in breast tumors through the overexpression of these cell surface protein-coding genes. Furthermore, a comparison was made between the genes found in this report and other genes associated with features clinically relevant for breast tumorigenesis. *Conclusions*. The expression profiling generated in this study, together with an integrative bioinformatics analysis, allowed us to identify putative targets for breast tumors.

## 1. Introduction

Breast tumors are among the most common fatal cancers, accounting for half of the total cancer deaths in women [1]. It has been shown that breast cancer is a very heterogeneous disease with four different molecular profiles based on the expression pattern of ERBB2, estrogen and progesterone receptors, and histological grade. These classes are also associated with distinct clinical outcomes and responses to therapy [2].

According to the American Society of Clinical Oncology [3], breast cancers express some additional markers that have been shown to be useful in clinic: CA15-3, CA27.29, carcinoembryonic antigen (CEA), urokinase plasminogen activator, and plasminogen activator inhibitor 1, among others. Several of these markers are cell surface transmembrane proteins, including ERBB2 and CEA. In addition, gene signatures identified by gene-expression profiling of breast tumors, such as the MammaPrint [4], which calculates a prognostic score for node-negative patients at stage I or II based on the expression of a pool of 70 genes, contain more than 10% of trans-membrane (TM) proteins believed to be at the cell surface [5].

Some of these cell surface markers are used as targets for therapies against breast cancer. Targeted therapies, such as lapatinib (Tykerb) [6], bevacizumab (Avastin) [7], and trastuzumab (Herceptin), are gaining special attention in cancer treatment as they act directly to cancer cells avoiding the destruction of healthy cells. Two of them are monoclonal antibodies that block directly or indirectly the activation of a trans-membrane receptor. Herceptin binds to the extracellular domain of ERBB2 and is used routinely in the treatment of patients with ERBB2 positive breast tumors. However, ERBB2 is just over expressed in 20–25% of invasive breast tumors [8, 9]. These facts emphasize the importance of exploring cell surface proteins in tumors to identify new tumor targets. Besides being targets for monoclonal antibodies that block their function, trans-membrane proteins can also be used to allow the entrance of drugs or radioactive material directly into cancer cells. 

A catalog of 3,702 genes coding for TM proteins located at the surface of human cells (human cell surfaceome) was generated by us through the use of bioinformatics approaches [5]. New targets for colorectal and glioblastoma (GBM) tumors have been identified using this collection of cell surface proteins. Here we explored the human cell surfaceome searching for breast tumors targets using a high-throughput quantitative real-time PCR (qPCR) in tumor samples and tumor cell lines. Furthermore, we compared our data with other expression profiles publically available taking into consideration some clinical features important for breast tumors.

## 2. Materials and Methods

### 2.1. Public Data

Breast cancer somatic mutations were obtained from Sjöblom et al. [10] and Wood et al. [11]. Homozygotic deletions and genomic amplifications in breast cancer were retrieved from Leary et al. [12]. The genomic coordinates for these alterations were determined through a local mapping using BLAT and crossed against surfaceome genes coordinates. Gene pathways were downloaded from KEGG (http://www.genome.jp/kegg/). To build the protein protein interaction (PPI) database, data from MINT (December 2007 version), BIOGRID (2.0.37), INTACT (January 2008 version), HPRD (September 2007 version), BIND (May 2006 version), and DIP (January 2008 version) were used. Data obtained exclusively from functional interactions (e.g., genetic interactions present in BIOGRID) and mass spectrometry-based methods were excluded.

### 2.2. Samples

The twelve breast tumor samples (ERBB2 negative) were obtained from the Tumor Tissue Biobank of the Medical and Research Center, A.C. Camargo Hospital, São Paulo, after local ethics committee approval. Human tumor breast cell lines (HCC1954, MCF7, MDA231, MDA435, MDA436, MDA468, and SK-BR 3) were obtained from American Type Culture Collection (ATCC) and cultured following ATCC instructions. A nontumorigenic and nontransformed breast cell line (HB4A) was obtained from reduction mammoplasty tissue [13]. Total RNA derived from 21 normal human tissues (thymus, prostate, fetal brain, trachea, skeletal muscle, fetal liver, uterus, small intestine, heart, bone marrow, kidney, stomach, liver, spleen, spinal cord, lung, testis, placenta, brain whole, breast, and colon) was purchased from Clontech.

### 2.3. Primer Design

Primers and the specific UPL probes were designed using the Universal ProbeLibrary Assay Design Center (http://qpcr.probefinder.com/organism.jsp) by automatically selecting an intron spanning assay. Rpl27 (NM_021574.2—ribosomal protein l27) and bcr (NM_021574.2—breakpoint cluster region) were used as the references genes. All primers and probes used are available in [5].

### 2.4. RNA Extraction, cDNA Synthesis, and Preamplification Reaction

 RNA extraction, cDNA synthesis, and pre-amplification reaction were performed as previously described in [5]. Briefly, total RNA was extracted by using Trizol (Invitrogen), and its integrity, checked by Bioanalyser (Agilent). SuperScript III First-Strand Synthesis SuperMix (Invitrogen) was used to synthesize cDNA from 200 ng of DNA-free RNA. For the pre-amplification reaction, one fourth of cDNA (synthesized as described above) was pooled with all primers (50 nM), 10 *μ*L of 2 X PreAmp Master Mix (Applied Biosystems) in a cycling program of 14 cycles of 95°C for 15 sec and 60°C for 4 min.

### 2.5. qPCR

The qPCR reactions for 573 genes were performed in 96.96 dynamic array chips (Fluidigm) as previously described [5] following the manufacturer's instructions. In summary, the assay inlets of the M96 Dynamic Array were loaded with 2 *μ*M forward primer, 2 *μ*M reverse primer, 1 *μ*M UPL probe, and 0.025% Tween 20 while each sample inlets was loaded with 2.5 *μ*L of PreAmp sample, 3.25 *μ*L of 2 X AB Universal TaqMan Master Mix (Applied Biosystems), and 0.32 *μ*L of 20 X DA Sample Loading Solution (Fluidigm). The cycling program used consisted of 2 min at 50°C, 10 min at 95°C followed by 35 cycles of 95°C for 15 sec, 70°C for 5 sec, and 1 min at 60°C. All reactions were performed in triplicates.

### 2.6. Data Processing of qPCR and Selection of Overexpressed Genes

The BioMark Gene Expression Data Analysis was used to obtain the CT values and to discriminate between high-quality and low-quality reactions. Low-quality reactions and CTs higher than 30 were excluded from these analyses and named as not available (NA). The 2^−ΔΔCT^ [14] method was used to obtain the relative gene expression values; normal whole breast (from Clontech) was used as a reference, and Rpl27 and bcr were used as reference genes. Genes were considered overexpressed in breast tumors if 3 or more primary breast tumors had a fold change higher than three times the standard deviation in all three replicates. Genes expressed in 5 or less normal tissues were considered restrictedly expressed. In order to be considered expressed, the gene/sample must have a fold change value higher than 0.1 in two qPCR experiments. Expression in fetal brain, fetal liver, brain, placenta, and testis was not considered in this analysis.

### 2.7. Comparative Analysis Using ONCOMINE

For the analysis of overexpression in other tumor types, the ONCOMINE website (http://www.oncomine.org) was used for searching individually (“Gene search”) the presence of each of the 77 overexpressed genes in the studies that compare “Cancer & Normal” with *P* value < 0.01. For the correlation analysis of over expression and prognosis, tumor grade and stage, we searched (“Profile Search”) genes over expressed (*P*-value < 0.01) on breast studies using the filters “Prognosis,” “Grade” and “Stage” existing at the ONCOMINE website (http://www.oncomine.org). Manual inspections of the studies were performed in others to select the most concordant (regarding samples types) in each category.

## 3. Results and Discussion

### 3.1. Genes Differentially Expressed in Breast Tumors

 Recently, we have generated a catalog of 3,702 genes that codes for trans-membrane proteins located at cell surface [5]. In that report, we further selected 573 cell surface genes as the most promising candidates for cancer diagnostics and therapy based on their expression profiling through the use of Massively Parallel Signature Sequencing (MPSS) and Serial Analysis of Gene Expression (SAGE) libraries. We validated this strategy by finding potential new human targets for GBM and colorectal tumors. Here, we analyzed the expression of these 573 genes in 12 primary breast tumors, 1 normal breast tissue, 7 breast tumor cell lines, and 1 normal breast cell line using a high-throughput qPCR platform. We also analyzed these 573 genes in a panel of 21 normal samples obtained from our previous publication [5].

 An ideal candidate for breast cancer diagnostics and therapy would be a gene product that is over expressed in breast tumors and expressed in a restricted pattern in normal tissues. Based on that, our qPCR data was analyzed for genes expressed in less than 6 normal tissues and overexpressed in at least 3 primary breast tumors when compared to normal breast tissue. We found two genes showing this expression pattern: C3orf57 and LRRC26. According to our data, the former is expressed in brain, prostate, and stomach while the latter in colon and prostate. C3orf57 (also known as ADMP), a putative G-protein coupled receptor, is also expressed in prostate epithelium and was shown to be downregulated by androgens in mice cells [15]. LRRC26, a leucine-rich repeat protein, has been previously shown to be increased in breast, prostate, colon, and pancreatic cancers. However, its subcellular localization is still a matter of debate in spite of the presence of a trans-membrane domain [16].

**Figure 1:**
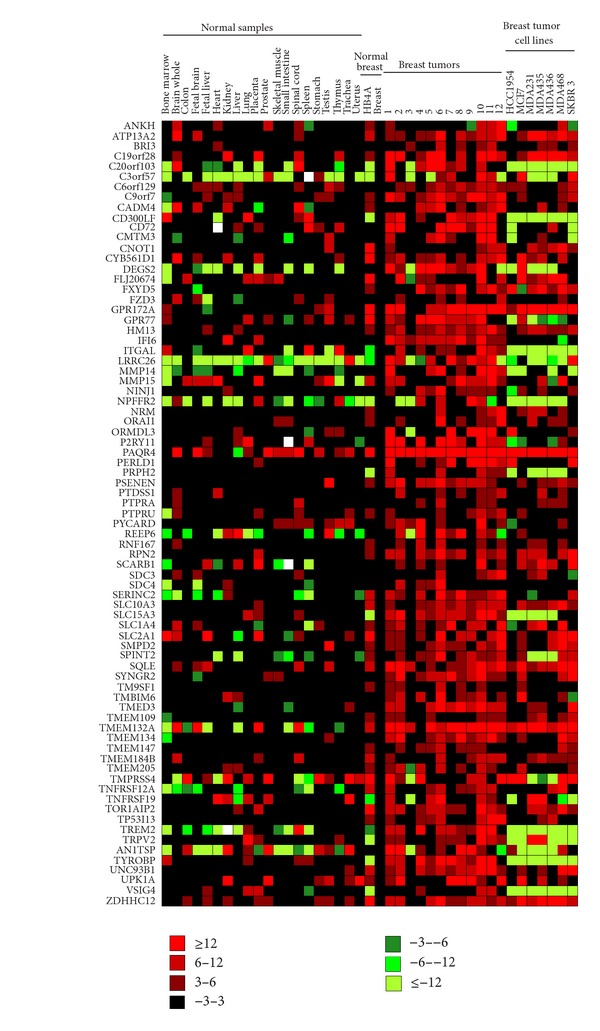
Expression profile of surfaceome genes in normal tissues, breast cancer cell lines, and breast tumor samples. Expression data for normal tissues was obtained from [5]. Colors represent the fold changes values obtained by qPCR (average of three qPCRs experiments) using normal breast as a reference sample and Rpl.27 as a reference gene. Red, green and black squares represent, respectively, genes over-expressed (fold changes ranging from 3 to 5.99, 6 to 11.99, and higher than 12 standard deviation values), genes downregulated (fold changes ranging from −3 to −5.99, −6 to −11.99, and lower than −12 standard deviation values), or genes equally expressed between the respective sample and reference. White squares represent noninformative data points.

 Irrespective of the expression pattern in normal tissues, we found 77 genes that were over expressed (in relation to normal breast) in primary breast tumors (Figure 1). Interestingly, these genes are enriched in specific genomic locations. Eight (CADM4, FXYD5, GPR77, PSENEN, SPINT2, TMEM147, TYROBP, and UPK1A) out of 77 genes are localized at chromosome 19q (*P*-value 0.006). In addition, an enrichment at the chromosome cytoband 1p35 (IFI6, PTPRU, SERINC2, and SLC2A1) is also observed (*P*-value 4.5E-4). Interestingly, this cytoband is associated with sporadic colorectal cancer, an Epstein-Barr virus integration site, and with some hormone-dependent syndromes such as serkal syndrome, mullerian aplasia, and hyperandrogenism (OMIM—http://www.ncbi.nlm.nih.gov/Omim/getmap.cgi?l132850). SLC2A1, also known as GLUT-1, is a glucose transporter, that is mutated in GBM and pancreatic tumor. Furthermore, it is expressed in various tumors, like oral squamous cell carcinoma [17], primary renal tumors [18], ovarian carcinoma [19], colorectal tumors [20], thyroid carcinomas [21], and gallbladder carcinomas [22]. Differential expression of this gene in breast tumors emphasizes the importance of glucose transporters in the glucose requirements in cancer cells. As we used only ERBB2 negative samples in this study, this gene accordingly was not present in our list of over expressed genes.

**Figure 2:**
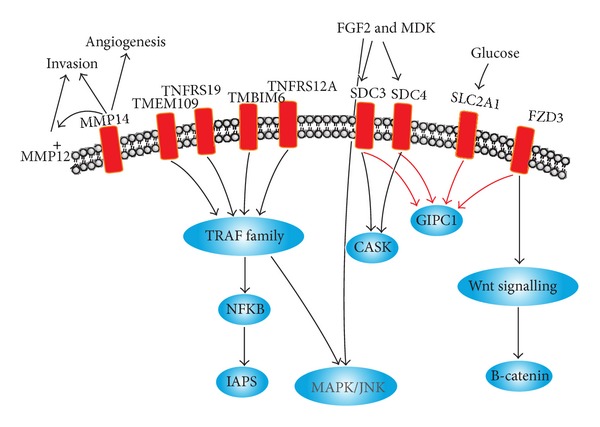
Schematic view of key pathways represented in the set of surfaceome genes overexpressed in breast tumors (see text for more details). Interactions represented by black arrows were derived from protein-protein interaction networks while interactions represented by red arrows were derived from either the literature or KEGG database.

 Next, we looked at protein-protein interaction (PPI) networks, KEGG, and the literature to find common interaction partners among differentially expressed surfaceome genes, as we did in [5] (Figure 2). Four genes (TNFRSF19, TNFRSF12A, TMEM109, and TMBIM6) interact with members of the TRAF family. TRAF proteins are TNF receptor-associated factors that serve as adapter proteins for a wide variety of cell surface receptors. They regulated the activation of many signal transduction pathways, such as JNK, ERK, and NF-*κ*B culminating in regulation of cell survival, apoptosis, and differentiation [23].

Furthermore, we found that GIPC1, a member of PDZ domain containing family that has already been detected as overexpressed in breast tumors [24], interacts with four genes of our list of overexpressed surfaceome genes (SDC3, SDC4, SLC2A1, and FDZ3). GIPC1 leads to an increase of TGF beta receptors and may interfere with TGF beta signaling [25]. SDC3 and SDC4 are syndecans that contain heparan and chondroitin sulfates chains that play a role in cell-cell and cell-matrix interactions [26]. Orend et al. [27] proposes that SDC4 may play an important role in the control of anchorage-dependent cellular proliferation of fibroblasts. The FZD3, a member of frizzled gene family, contributes to the Wnt signaling pathway which leads to an increase of the beta-catenin in the nucleus promoting specific gene expression [28]. MPSS analysis also confirms that this gene is over expressed in breast tumors (data not shown).

### 3.2. Further Validation of Overexpressed Genes

To gain more information on the surfaceome genes over-expressed in breast tumors, a comparison with expression data from other sources was performed. We first compared our list of genes with our previous study [5] in which the expression of the same set of genes was evaluated in colorectal tumors and GBM. Seven (GPR172A, IFI6, NPFFR2, REEP6, SLC2A1, SQLE, and TNFRSF12A) and 13 (CD300LF, CD72, CMTM3, GPR172A, MMP14, P2RY11, SERINC2, TMEM134, TNFRSF12A, TNFRSF19, TYROBP, VSIG4, and ZDHHC12) genes are also over expressed in colorectal tumors and GBM, respectively. Among them, MMP14, a membrane-bound matrix metalloprotease (MMP), seems one of the most interesting. It has been associated with tumorigenesis, metastasis, and angiogenesis [29–31] and activates pro-MMP2 [32, 33], a MMP known to be critically involved in tumor spreading. Recently, Devy et al. [34] identified a human antibody that is a potent and highly selective inhibitor of MMP-14. It reduces the incidence of metastases in orthotopic mouse model, displayed anti-invasive activity, and inhibited angiogenesis. The authors propose that this inhibitor could be used for the treatment of solid tumors.

 Moreover, we compared the expression of our set of overexpressed surfaceome genes in breast tumors to 16 other tumor types, including breast, using microarray data from studies available at ONCOMINE (http://www.oncomine.org). As shown in Table 1, we confirmed that more than a half (57,1%) of our genes are overexpressed in at least another study using breast tumors. The other part, however, most probably reflects different experimental sensitivities between qPCR and microarray as well as different breast tumor types. In addition, 53 out of 77 are over expressed in more than 4 tumor types.

It is also important to know whether any of our over expressed surfaceome genes is associated with a clinically relevant characteristic. Genes that are overexpressed (*P* values < 0.01) in studies that compared samples with distinct prognosis, grades, or stages of breast tumors were selected by looking at the ONCOMINE dataset (Table 2). Thirty-seven out of 77 genes were expressed (in at least one study) in breast tumors of higher grades. Concerning the staging of breast cancer using the TNM classification, six genes (FXYD5, MMP15, PTPRU, SQLE, TM9SF1, and TNFRS12A) are overexpressed in samples with increased spread to lymph nodes, while one gene (TYROBP) is over expressed when breast tumors samples without and with metastasis were compared. Twenty-five out of 77 genes are over expressed in samples derived from patients with high mortality rate within 5 years while 24 out of 77 genes are over expressed in samples derived from patients that relapsed within 5 years. 

 The result of these comparisons strongly suggests that the set of surfaceome genes generated in this report is enriched with genes important for breast tumor. 

### 3.3. Surfaceome Genes and Genetic Alterations in Breast Tumors

**Figure 3:**
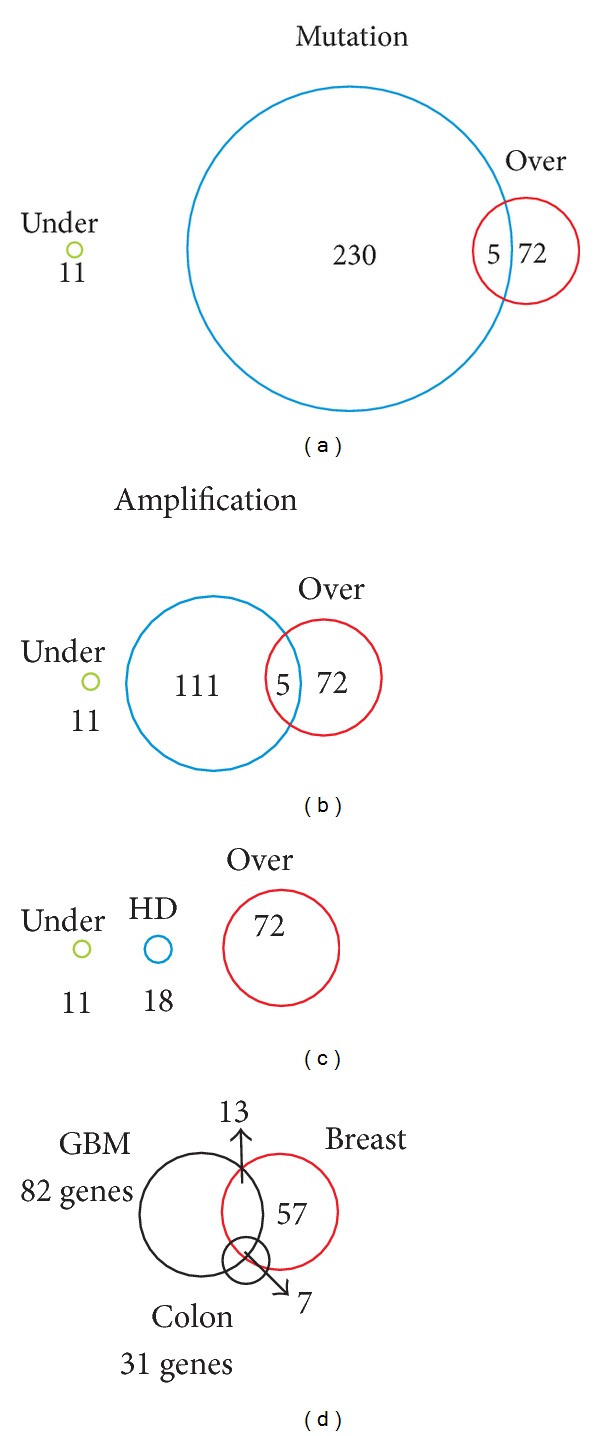
Comparison of over- and underexpressed genes in breast tumors with previous studies that identified genes with somatic mutation (a), chromosome amplification (b), or homozygous deletions (c) in breast tumors. (d) Comparison of surfaceome genes over-expressed in breast tumors with surfaceome genes over-expressed in GBM and colorectal tumors [5].

 We also analyzed whether the human surfaceome is associated with genes commonly altered in breast tumors based on previously published data [35] that investigated the presence of homozygous deletion, chromosome amplification, and somatic mutation in breast tumor samples. Eighteen surfaceome genes (BVES, C9orf11, CDH3, FAT, LINGO2, LRRC19, MTNR1A, OR4C6, OR4S2, PCDH8, POPDC3, PTCHD3, PTPLAD2, ROBO2, SGPP1, SLITRK1, TEK, and TLR3) were found previously to be homozygously deleted in breast tumors. Not surprisingly, none of them are present in our list of over expressed genes. In contrast, 116 surfaceome genes are localized in genomic regions that contain focal amplifications in cancer (Figure 3 and See Table S1 in Supplementary Material available online at http://dx.doi.org/10.1155/2013/976816). GPR172A, ORMDL3, PERLD1, PRPH2, and TREM2 are presented in our list of over expressed genes. Among them, PERLD1 is located in a chromosome 17 locus together with well known oncogenes (MYC, ERBB2, MET, and FGFR2 among others) that are frequently amplified in gastric and breast cancers [36]. However, the subcellular localization of some of these genes is still a matter of debate. 

 Two hundred and thirty-five surfaceome genes (6,35% of the total surfaceome collection) have been previously identified as having somatic mutations in breast tumor [10, 11]. Four of them (C19orf28, HM13, ITGAL, and MMP15) are also over expressed in breast tumors according to our analysis (Table S1).

## 4. Conclusions

The expression profiling generated in this work, together with an integrative analysis using other genomics information, allows some considerations. First, like in GBM [5], signaling through members of the TRAF family seems to be important in breast tumorigenesis. In particular the gene TNFRSF12A (also known as TWEAK receptor) seems to be critical as it was also detected as overexpressed in GBM and colorectal tumors samples [5]. We confirmed that this gene is significantly overexpressed in breast tumors and seems to be associated with local invasiveness [37]. In addition, MMP15, a metalloproteinase that was found over expressed in our samples, is also over expressed in samples with important clinical features such as higher grades, positive lymph nodes, and poor prognosis (Table 2). It is important to note that this study focus on expression of cell surface gene products thus, well-known cancer genes products localized at cytoplasm or nucleus were not detected in this dataset. In addition, we focused our analysis and discussion on genes that have been previously associated with tumorigenesis and seem to have clinical relevance. Although presented evidence suggests that the overexpressed set of genes is enriched with genes important for breast tumorigenesis, additional studies are needed to establish any clinical applicability. Likewise, additional analyses are also required to uncover the function of the remaining genes that were not found to be associated with cancer and that were not explored in this work.

## Supplementary Material

Supplementary Table show surfaceome genes that contains mutations, gene amplication or homozygotic deletion (according to previously published data) in breast tumors.

## Figures and Tables

**Table 1:** Evaluation of breast tumor overexpressed genes in other tumor types according to ONCOMINE.

Gene	Tumors														
	Bladder	Bone marrow	Brain	Breast	Colon	Esophagus	Head and neck	Kidney	Leukemia	Liver	Lung	Ovary	Pancreas	Prostate	Skin
ANKH	—	—	—	—	*∙*	—	*∙*	*∙*	*∙*	—	—	—	*∙*	—	—
ATP13A2	*∙*	—	—	—	—	*∙*	*∙*	—	—	—	—	—	—	*∙*	*∙*
BRI3	—	—	*∙*	*∙*	—	—	*∙*	—	—	—	—	—	—	—	—
C19orf28	*∙*	*∙*	—	*∙*	—	—	—	—	—	—	—	*∙*	*∙*	*∙*	*∙*
C20orf103	—	—	—	*∙*	*∙*	—	*∙*	—	—	—	—	—	*∙*	*∙*	—
C6orf129	—	—	*∙*	*∙*	—	—	*∙*	—	—	—	—	*∙*	—	*∙*	—
C9orf7	*∙*	—	*∙*	*∙*	—	—	—	—	—	—	—	*∙*	—	*∙*	*∙*
C3orf57	—	—	—	—	—	—	—	—	—	—	—	—	—	—	—
CADM4	—	—	*∙*	—	—	—	—	—	—	*∙*	*∙*	—	—	—	*∙*
CD300LF	—	—	—	*∙*	—	—	—	—	—	—	—	—	—	—	—
CD72	*∙*	—	—	*∙*	—	—	*∙*	—	—	—	*∙*	—	—	—	—
CMTM3	—	—	*∙*	*∙*	—	—	—	*∙*	—	—	—	—	—	*∙*	—
CNOT1	*∙*	—	*∙*	*∙*	*∙*	—	—	—	*∙*	—	*∙*	—	—	*∙*	—
CYB561D1	—	*∙*	—	—	—	—	—	—	—	*∙*	—	—	—	*∙*	—
DEGS2	—	—	—	—	—	—	—	—	*∙*	—	—	—	—	—	—
FLJ20674	*∙*	—	*∙*	*∙*	—	—	—	—	—	—	—	—	—	—	—
FXYD5	*∙*	—	*∙*	*∙*	*∙*	—	—	*∙*	—	—	—	*∙*	*∙*	—	—
FZD3	—	—	—	—	—	—	*∙*	—	—	—	*∙*	—	—	—	*∙*
GPR172A	*∙*	—	*∙*	*∙*	*∙*	*∙*	*∙*	—	—	*∙*	*∙*	*∙*	*∙*	*∙*	*∙*
GPR77	—	—	—	—	—	—	*∙*	—	—	—	—	—	—	—	*∙*
HM13	—	—	*∙*	*∙*	*∙*	—	—	—	—	—	—	*∙*	*∙*	—	—
IFI6	*∙*	*∙*	*∙*	*∙*	—	*∙*	*∙*	*∙*	—	*∙*	—	—	*∙*	*∙*	*∙*
ITGAL	—	—	*∙*	—	—	—	—	—	—	—	—	—	—	—	—
LRRC26	—	—	—	—	—	—	—	—	—	—	—	—	—	—	—
MMP14	*∙*	—	*∙*	*∙*	*∙*	*∙*	*∙*	—	—	*∙*	*∙*	*∙*	*∙*	*∙*	*∙*
MMP15	*∙*	—	*∙*	—	—	*∙*	—	—	—	—	*∙*	*∙*	—	*∙*	—
NINJ1	—	—	*∙*	—	*∙*	—	*∙*	*∙*	—	—	—	—	—	—	*∙*
NPFFR2	—	—	—	*∙*	—	—	—	—	—	—	—	—	—	—	—
NRM	—	—	*∙*	*∙*	—	—	—	—	*∙*	*∙*	*∙*	—	—	*∙*	*∙*
ORAI1	—	—	*∙*	—	*∙*	*∙*	—	—	—	—	*∙*	—	—	*∙*	—
ORMDL3	—	*∙*	*∙*	—	*∙*	—	—	*∙*	—	—	—	—	—	—	—
P2RY11	—	—	*∙*	—	—	—	—	—	—	—	—	*∙*	—	*∙*	*∙*
PAQR4	*∙*	—	—	*∙*	—	—	*∙*	—	*∙*	*∙*	*∙*	*∙*	*∙*	—	*∙*
PERLD1	*∙*	—	—	*∙*	—	—	—	*∙*	*∙*	—	—	*∙*	—	—	—
PRPH2	—	—	—	—	—	—	—	—	—	—	—	—	*∙*	—	—
PSENEN	—	—	—	—	—	—	—	—	—	*∙*	—	—	—	—	—
PTDSS1	*∙*	*∙*	*∙*	*∙*	*∙*	*∙*	*∙*	—	*∙*	*∙*	*∙*	*∙*	—	*∙*	*∙*
PTPRA	*∙*	*∙*	*∙*	*∙*	—	*∙*	—	*∙*	*∙*	—	*∙*	—	*∙*	—	*∙*
PTPRU	*∙*	—	—	—	—	*∙*	—	—	*∙*	—	*∙*	*∙*	—	*∙*	*∙*
PYCARD	*∙*	—	*∙*	*∙*	—	—	*∙*	*∙*	—	—	—	—	—	—	—
REEP6	—	*∙*	—	*∙*	—	—	—	*∙*	*∙*	—	—	—	—	—	—
RNF167	—	*∙*	—	—	—	—	—	—	—	—	*∙*	—	—	*∙*	—
RPN2	*∙*	—	*∙*	*∙*	*∙*	*∙*	*∙*	—	*∙*	—	*∙*	*∙*	*∙*	—	*∙*
SCARB1	*∙*	*∙*	—	—	—	*∙*	*∙*	*∙*	—	—	*∙*	—	—	*∙*	*∙*
SDC3	—	—	*∙*	*∙*	—	*∙*	*∙*	*∙*	—	—	*∙*	—	—	*∙*	*∙*
SDC4	*∙*	—	—	—	—	*∙*	*∙*	—	—	—	—	*∙*	—	*∙*	—
SERINC2	—	—	*∙*	*∙*	—	—	—	—	—	*∙*	*∙*	*∙*	*∙*	*∙*	—
SLC10A3	*∙*	*∙*	*∙*	*∙*	—	—	—	—	—	*∙*	—	—	—	—	—
SLC15A3	*∙*	—	*∙*	*∙*	—	*∙*	*∙*	*∙*	—	—	—	—	—	—	—
SLC1A4	—	—	*∙*	*∙*	*∙*	—	*∙*	*∙*	*∙*	*∙*	*∙*	*∙*	—	*∙*	—
SLC2A1	*∙*	*∙*	—	*∙*	*∙*	—	*∙*	*∙*	*∙*	—	*∙*	*∙*	*∙*	—	—
SMPD2	*∙*	—	*∙*	—	—	—	—	*∙*	—	*∙*	*∙*	*∙*	—	—	*∙*
SPINT2	*∙*	—	—	*∙*	—	—	*∙*	*∙*	—	—	*∙*	*∙*	*∙*	*∙*	—
SQLE	*∙*	—	*∙*	*∙*	—	—	*∙*	—	—	*∙*	*∙*	*∙*	—	*∙*	*∙*
SYNGR2	*∙*	—	—	*∙*	*∙*	—	—	*∙*	—	—	*∙*	*∙*	*∙*	*∙*	—
TM9SF1	*∙*	—	*∙*	—	—	—	*∙*	—	*∙*	*∙*	*∙*	*∙*	—	*∙*	—
TMBIM6	*∙*	—	*∙*	—	—	—	—	—	—	—	*∙*	*∙*	*∙*	*∙*	—
TMED3	*∙*	*∙*	*∙*	—	*∙*	—	—	*∙*	*∙*	—	*∙*	—	—	*∙*	*∙*
TMEM109	—	*∙*	—	*∙*	—	—	—	—	*∙*	—	—	—	—	—	*∙*
TMEM132A	*∙*	—	—	*∙*	—	—	*∙*	—	—	—	*∙*	*∙*	*∙*	*∙*	—
TMEM134	*∙*	—	*∙*	—	—	—	—	—	—	—	*∙*	*∙*	—	*∙*	—
TMEM147	—	—	*∙*	—	*∙*	—	—	—	—	*∙*	*∙*	*∙*	—	*∙*	—
TMEM184B	*∙*	—	—	—	—	*∙*	*∙*	*∙*	*∙*	*∙*	*∙*	*∙*	*∙*	*∙*	*∙*
TMEM205	—	—	—	—	—	—	—	—	—	—	—	—	—	—	—
TMPRSS4	—	—	—	—	*∙*	—	—	—	—	—	*∙*	*∙*	—	—	—
TNFRSF12A	—	*∙*	*∙*	—	*∙*	*∙*	*∙*	*∙*	—	—	*∙*	*∙*	—	—	*∙*
TNFRSF19	—	—	*∙*	—	—	—	—	—	—	*∙*	—	—	—	*∙*	—
TOR1AIP2	—	*∙*	*∙*	*∙*	—	—	—	—	—	*∙*	—	—	—	—	—
TP53I13	—	—	*∙*	*∙*	—	—	—	—	—	—	—	—	—	—	—
TREM2	*∙*	—	*∙*	*∙*	—	*∙*	*∙*	*∙*	—	*∙*	—	—	*∙*	—	*∙*
TRPV2	—	—	—	*∙*	—	*∙*	—	—	—	—	—	—	—	*∙*	—
TSPAN1	*∙*	—	—	*∙*	—	*∙*	*∙*	—	—	—	—	*∙*	*∙*	*∙*	—
TYROBP	—	—	*∙*	*∙*	*∙*	—	*∙*	*∙*	—	—	—	—	—	—	—
UNC93B1	*∙*	*∙*	*∙*	*∙*	—	—	—	—	*∙*	—	—	*∙*	—	—	—
UPK1A	—	—	—	*∙*	—	—	—	—	—	—	—	—	—	—	—
VSIG4	—	—	*∙*	*∙*	—	*∙*	*∙*	*∙*	*∙*	—	—	—	—	—	—
ZDHHC12	—	—	*∙*	*∙*	—	—	—	—	—	—	—	*∙*	—	—	—

*∙*: means overexpression in a given tumor in at least one ONCOMINE study (*P* < 0.01).

**Table 2:** Evaluation of breast cell surface overexpressed genes according to ONCOMINE studies of grade, stage, and prognosis in breast tumors.

																Stage^2^											Prognosis									
	Grade^1^															T			N							M		Survival^3^				Disease-free survival^4^				
	Bittner	Chin	Desmedt	Farmer	Finak	Ginestier	Hess	Kreike	Ma	Miller	Pollack	Sorlie	Sotiriou	Sotiriou	Zhao	Bittner	Sorlie	Sorlie	Bittner	Hess	Sorlie	Sorlie	Sotiriou	Turashvili	Yu	Sorlie	Bild	Desmedt	Pawitan	Sorlie	VandeVijver	Ma	Wang	Wang	Van de Vijver	VandeVijver
SQLE	X	X	X	—	—	X	X	X	—	X	—	—	—	X	X	—	—	—	X	—	—	—	—	—	—	—	—	—	X	—	X	—	X	X	X	X
CNOT1	X	X	X	X	—	X	X	—	—	X	—	—	—	X	—	—	—	—	—	—	—	—	—	—	—	—	—	—	X	—	X	—	X	X	—	—
TMEM132A	X	—	X	X	—	X	X	—	—	X	—	—	—	X	—	—	—	—	—	—	—	—	—	—	—	—	—	—	X	—	—	X	—	X	X	—
PTDSS1	X	—	X	—	—	X	X	—	—	X	—	X	—	X	—	—	—	—	—	—	—	—	—	—	—	—	—	—	—	X	X	—	—	—	X	X
RPN2	X	—	X	X	X	X	X	—	—	X	—	—	—	—	—	—	—	—	—	—	—	—	—	—	—	—	—	—	—	—	X	—	—	—	X	X
SLC2A1	X	—	X	X	—	X	—	—	—	X	—	—	—	X	X	—	—	—	—	—	—	—	—	—	—	—	—	—	—	—	X	—	—	—	X	X
GPR172A	X	—	X	X	—	X	X	—	—	X	—	—	—	X	—	—	—	—	—	—	—	—	—	—	—	—	—	—	X	—	—	—	—	—	—	—
MMP15	—	—	—	X	—	—	X	—	—	—	—	—	—	X	—	—	—	—	—	—	—	—	X	—	—	—	—	—	X	—	X	—	—	—	X	X
UNC93B1	X	—	—	—	—	X	—	—	—	—	—	—	—	X	—	—	—	—	—	—	—	—	—	—	—	—	—	—	—	—	X	—	—	—	X	X
C6orf129	X	—	—	—	—	X	—	—	X	—	—	—	—	—	X	—	—	—	—	—	—	—	—	—	—	—	—	—	X	—	—	—	—	—	—	—
NRM	X	—	—	—	—	X	—	—	X	—	—	—	—	—	—	—	—	—	—	—	—	—	—	—	—	—	—	—	—	—	X	—	—	—	X	—
SLC10A3	X	—	—	—	—	—	—	—	—	X	—	—	—	—	—	—	—	—	—	—	—	—	—	—	—	—	—	—	—	—	X	—	—	—	X	X
CD72	X	—	X	—	—	—	—	—	X	—	—	—	X	—	—	—	—	—	—	—	—	—	—	—	—	—	—	—	—	—	—	—	—	—	—	—
TNFRSF12A	X	—	—	—	—	X	—	—	—	X	—	—	—	—	—	—	—	—	—	—	—	—	X	—	—	—	—	—	—	—	—	—	—	—	—	—
IFI6	X	—	—	—	—	—	—	X	—	—	—	—	—	—	—	—	—	—	—	—	—	—	—	—	—	—	—	—	—	—	—	—	—	—	X	X
BRI3	X	—	—	—	—	—	—	—	—	—	—	—	—	—	X	—	—	—	—	—	—	—	—	—	—	—	—	—	—	—	X	—	—	—	X	—
SCARB1	X	—	—	—	—	—	—	—	—	—	—	—	—	—	—	—	—	—	—	—	—	—	—	—	—	—	—	—	—	—	X	—	—	—	X	X
PERLD1	—	X	—	—	—	—	X	—	—	X	—	—	—	X	—	—	—	—	—	—	—	—	—	—	—	—	—	—	—	—	—	—	—	—	—	—
TRPV2	—	—	—	—	—	X	—	—	—	—	—	—	—	—	—	—	—	—	—	—	—	—	—	—	—	—	—	—	—	—	X	X	—	—	X	—
ORMDL3	X	X	—	—	—	—	—	—	—	—	—	—	—	—	—	—	—	—	—	—	—	—	—	—	—	—	—	—	X	—	—	—	—	—	—	—
C19orf28	X	—	—	—	—	X	—	—	—	—	—	—	—	—	X	—	—	—	—	—	—	—	—	—	—	—	—	—	—	—	—	—	—	—	—	—
PAQR4	X	—	—	—	—	—	—	—	—	X	—	—	—	—	—	—	—	—	—	—	—	—	—	—	—	—	—	—	X	—	—	—	—	—	—	—
TM9SF1	—	—	—	X	—	—	—	—	—	—	—	—	—	—	—	—	—	—	X	—	—	—	—	—	—	—	—	—	X	—	—	—	—	—	—	—
MMP14	—	—	—	—	—	X	—	—	—	—	—	—	—	—	—	—	—	—	—	—	—	—	—	—	—	—	—	—	—	—	X	—	X	—	—	—
PTPRU	—	—	—	—	—	—	—	—	X	—	—	—	—	—	—	—	—	—	—	—	—	—	X	—	—	—	—	—	X	—	—	—	—	—	—	—
TMEM109	X	—	—	X	—	—	—	—	—	—	—	—	—	—	—	—	—	—	—	—	—	—	—	—	—	—	—	—	—	—	—	—	—	—	—	—
P2RY11	—	—	—	X	—	X	—	—	—	—	—	—	—	—	—	—	—	—	—	—	—	—	—	—	—	—	—	—	—	—	—	—	—	—	—	—
UPK1A	—	—	—	X	—	—	—	—	—	—	—	—	—	—	—	—	—	—	—	—	—	—	—	—	—	—	—	—	—	—	—	X	—	—	—	—
CADM4	—	—	—	—	—	X	—	—	—	—	—	—	—	—	—	—	—	—	—	—	—	—	—	—	—	—	—	—	X	—	—	—	—	—	—	—
TYROBP	—	—	—	—	—	—	—	—	—	—	—	X	—	—	—	—	—	—	—	—	—	—	—	—	—	X	—	—	—	—	—	—	—	—	—	—
FXYD5	—	—	—	—	—	—	—	—	—	—	—	—	—	—	—	—	—	—	—	—	—	—	—	—	X	—	—	—	—	—	X	—	—	—	—	—
SPINT2	—	—	—	—	—	—	—	—	—	—	—	—	—	—	—	—	—	—	—	—	—	—	—	—	—	—	—	—	—	—	—	—	—	—	X	X
SYNGR2	—	—	—	—	—	—	—	—	—	—	—	—	—	—	—	—	—	—	—	—	—	—	—	—	—	—	—	—	—	—	—	—	—	—	X	X
TMPRSS4	—	—	—	—	—	—	—	—	—	—	—	—	—	—	—	—	—	—	—	—	—	—	—	—	—	—	—	—	—	—	—	—	—	—	X	X
ORAI1	X	—	—	—	—	—	—	—	—	—	—	—	—	—	—	—	—	—	—	—	—	—	—	—	—	—	—	—	—	—	—	—	—	—	—	—
ZDHHC12	X	—	—	—	—	—	—	—	—	—	—	—	—	—	—	—	—	—	—	—	—	—	—	—	—	—	—	—	—	—	—	—	—	—	—	—
SDC4	—	X	—	—	—	—	—	—	—	—	—	—	—	—	—	—	—	—	—	—	—	—	—	—	—	—	—	—	—	—	—	—	—	—	—	—
SDC3	—	—	—	—	—	X	—	—	—	—	—	—	—	—	—	—	—	—	—	—	—	—	—	—	—	—	—	—	—	—	—	—	—	—	—	—
TMEM184B	—	—	—	—	—	X	—	—	—	—	—	—	—	—	—	—	—	—	—	—	—	—	—	—	—	—	—	—	—	—	—	—	—	—	—	—
TOR1AIP2	—	—	—	—	—	—	—	—	X	—	—	—	—	—	—	—	—	—	—	—	—	—	—	—	—	—	—	—	—	—	—	—	—	—	—	—
TMEM147	—	—	—	—	—	—	—	—	—	—	—	—	—	—	X	—	—	—	—	—	—	—	—	—	—	—	—	—	—	—	—	—	—	—	—	—
TMED3	—	—	—	—	—	—	—	—	—	—	—	—	—	—	—	—	—	—	—	—	—	—	—	—	—	—	—	X	—	—	—	—	—	—	—	—
TREM2	—	—	—	—	—	—	—	—	—	—	—	—	—	—	—	—	—	—	—	—	—	—	—	—	—	—	—	—	X	—	—	—	—	—	—	—
SLC15A3	—	—	—	—	—	—	—	—	—	—	—	—	—	—	—	—	—	—	—	—	—	—	—	—	—	—	—	—	—	—	X	—	—	—	—	—
VSIG4	—	—	—	—	—	—	—	—	—	—	—	—	—	—	—	—	—	—	—	—	—	—	—	—	—	—	—	—	—	—	X	—	—	—	—	—
ANKH	—	—	—	—	—	—	—	—	—	—	—	—	—	—	—	—	—	—	—	—	—	—	—	—	—	—	—	—	—	—	—	X	—	—	—	—
ATP13A2	—	—	—	—	—	—	—	—	—	—	—	—	—	—	—	—	—	—	—	—	—	—	—	—	—	—	—	—	—	—	—	X	—	—	—	—
DEGS2	—	—	—	—	—	—	—	—	—	—	—	—	—	—	—	—	—	—	—	—	—	—	—	—	—	—	—	—	—	—	—	X	—	—	—	—
TNFRSF19	—	—	—	—	—	—	—	—	—	—	—	—	—	—	—	—	—	—	—	—	—	—	—	—	—	—	—	—	—	—	—	X	—	—	—	—
TMEM134	—	—	—	—	—	—	—	—	—	—	—	—	—	—	—	—	—	—	—	—	—	—	—	—	—	—	—	—	—	—	—	—	—	X	—	—

^1^Compares lower with higher grade samples.

^2^Compares samples derived from Tn with Tn + x (T); Nx with Nx + 1 (N); and Mx with Mx + 1 (M).

^3^Compares  samples derived from live and dead patients within 5 years.

^4^Compares samples derived from patients without and with relapse/recurrence in 5 years.

X: means overexpression in a given study (*P* < 0.01).

## Abbreviations

GBM: Glioblastoma
MPSS: Massively Parallel Signature Sequencing
qPCR: Quantitative Real-Time Polymerase Chain Reaction
SAGE: Serial Analysis of Gene Expression
TM: Transmembrane.
