# Supplementary material for: The Human Cell Surfaceome of Breast Tumors
Source: Biomed Res Int. 2013 Sep 9;2013:976816. doi: 10.1155/2013/976816 (PMC3781997; doi:10.1155/2013/976816)
Supplement: Supplementary file 1 — Supplementary Table show surfaceome genes that contains mutations, gene amplication or homozygotic deletion (according to previously published data) in breast tumors. [file 976816.f1.pdf]

TABLE S1.

Surfaceome genes that contain Mutations, Amplifications and Homozygotic Deletions (HD) in breast tumors

Gene	Mutation[1]	Amplification[2]	HD[2]
ABCA12	Y	-	-
ABCA3	Y	-	-
ABCA4	Y	-	-
ABCC8	-	Y	-
ADAM18	-	Y	-
ADAM2	-	Y	-
ADAM32	-	Y	-
ADAM9	-	Y	-
ADRA1A	Y	-	-
ADRB3	-	Y	-
ALCAM	Y	-	-
ALKBH5	-	Y	-
AMFR	Y	-	-
AMIGO1	Y	-	-
ANO1	-	Y	-
APOL1	Y	-	-
AQP8	Y	-	-
ASGR1	Y	-	-
ASTN2	Y	-	-
ATCAY	Y	-	-
ATG9A	Y	-	-
ATP12A	Y	-	-
ATP2A3	Y	-	-
ATP2C2	Y	-	-
ATP6V0B	Y	-	-
ATP8B1	Y	-	-
ATP8B4	Y	-	-
AVPR2	Y	-	-
BAI1	Y	-	-
BEST3	-	Y	-
BOC	Y	-	-
BVES	-	-	Y
C10orf54	Y	-	-
C14orf100	Y	-	-
C14orf101	Y	-	-
C14orf180	-	Y	-

C16orf58	Y	-	-
C18orf19	Y	-	-
C19orf12	-	Y	-
C19orf28	Y	-	-
C1orf2	Y	-	-
C1orf32	-	Y	-
C6orf174	Y	-	-
C9orf11	-	-	Y
CACNA1B	Y	-	-
CACNA1F	Y	-	-
CACNA1G	Y	-	-
CACNA1H	Y	Y	-
CACNA1I	Y	-	-
CATSPERB	Y	-	-
CCDC136	Y	-	-
CCR7	-	Y	-
CD2	Y	-	-
CD74	Y	-	-
CD97	Y	-	-
CDH10	Y	-	-
CDH20	Y	-	-
CDH26	-	Y	-
CDH3	-	-	Y
CDH8	Y	-	-
CDIPT	Y	-	-
CDON	Y	-	-
CHRND	Y	-	-
CLCA2	Y	-	-
CLCN1	Y	-	-
CLCN2	Y	-	-
CLCN7	-	Y	-
CLDN14	-	Y	-
CLPTM1L	Y	-	-
CMTM5	-	Y	-
CNGA3	-	Y	-
CNNM4	Y	-	-
CSF1R	Y	-	-
CXADR	-	Y	-
CXCR5	Y	-	-
CYHR1	-	Y	-
DC2	Y	-	-
DLK2	-	Y	-
DPY19L3	-	Y	-
DUOX1	Y	-	-

DYSF	Y	-	-
EDNRA	Y	-	-
ELOVL5	-	Y	-
EMR2	-	Y	-
ENTPD7	Y	-	-
EPHA8	Y	-	-
EPHB3	Y	-	-
ERBB2	-	Y	-
ERBB4	Y	-	-
EREG	Y	-	-
EVI2A	Y	-	-
EVI2B	Y	-	-
F2RL3	-	Y	-
FAM134B	Y	-	-
FAM171A1	Y	-	-
FANCM	Y	-	-
FAR1	-	Y	-
FAT	-	-	Y
FCRL3	Y	-	-
FCRL5	Y	-	-
FER1L3	Y	-	-
FGFR1	Y	Y	-
FGFR2	Y	-	-
FLJ46321	Y	-	-
FNDC3B	Y	-	-
GABRA4	Y	-	-
GABRP	Y	-	-
GFRAL	Y	-	-
GJA8	Y	-	-
GJB1	Y	-	-
GJD3	-	Y	-
GP5	Y	-	-
GPAA1	-	Y	-
GPNMB	Y	-	-
GPR111	Y	-	-
GPR115	Y	-	-
GPR124	-	Y	-
GPR172A	-	Y	-
GPR180	Y	-	-
GPR45	Y	-	-
GPR83	-	Y	-
GRAMD4	Y	-	-
GRIK2	Y	-	-
GRIK3	Y	-	-

GRIN2C	Y	-	-
GRIN2D	Y	-	-
GRINA	-	Y	-
GRM6	Y	-	-
GUCY2F	Y	-	-
HAS2	-	Y	-
HEPACAM2	Y	-	-
HLA-A	-	Y	-
HM13	Y	-	-
HRH3	-	Y	-
HTR2A	-	Y	-
ICAM5	Y	-	-
IL13RA1	-	Y	-
IL1RAPL2	Y	-	-
IL7R	Y	-	-
INPP4A	-	Y	-
ITGA9	Y	-	-
ITGAE	Y	-	-
ITGAL	Y	-	-
ITPR1	Y	-	-
KCNA5	Y	-	-
KCNC2	Y	-	-
KCNG1	-	Y	-
KCNJ1	Y	-	-
KCNJ11	-	Y	-
KCNJ15	Y	-	-
KCNMB4	-	Y	-
KCNT2	Y	-	-
KIAA1161	Y	Y	-
KIAA1324	Y	-	-
KIR2DL1	Y	-	-
KIR3DL1	Y	-	-
LHCGR	Y	-	-
LINGO2	-	-	Y
LMF2	Y	-	-
LRBA	Y	-	-
LRRC19	-	-	Y
LRRC24	-	Y	-
LY6G6F	Y	-	-
MAGEH1	-	Y	-
MAMDC4	Y	-	-
MBTPS2	Y	-	-
MCAM	Y	-	-
MCOLN1	Y	-	-

MFSD3	-	Y	-
MFSD9	Y	-	-
MGC35440	-	Y	-
MMP15	Y	-	-
MOSPD1	Y	Y	-
MPDU1	Y	-	-
MRGPRD	-	Y	-
MRGPRF	-	Y	-
MTNR1A	-	-	Y
NALCN	Y	-	-
NCR2	-	Y	-
NFASC	Y	-	-
NKAIN4	-	Y	-
NOX5	Y	-	-
NPBWR1	Y	-	-
NRCAM	Y	-	-
NRM	Y	-	-
NTRK3	Y	-	-
OCA2	Y	-	-
ODZ1	Y	-	-
OPRS1	-	Y	-
OR10A2	Y	-	-
OR10H4	Y	-	-
OR10Q1	-	Y	-
OR10W1	-	Y	-
OR12D3	Y	-	-
OR1I1	-	Y	-
OR1J2	Y	-	-
OR1N1	Y	-	-
OR1S1	Y	Y	-
OR1S2	-	Y	-
OR2M4	Y	-	-
OR2W3	Y	-	-
OR2W5	Y	-	-
OR4C6	-	-	Y
OR4D2	Y	-	-
OR4S2	-	-	Y
OR52A1	Y	-	-
OR52H1	Y	-	-
OR56A1	Y	-	-
OR5B12	-	Y	-
OR5B17	-	Y	-
OR5B2	-	Y	-
OR5B21	-	Y	-

OR5B3	-	Y	-
OR5H1	Y	-	-
OR5J2	Y	-	-
OR5M11	Y	-	-
OR6Q1	-	Y	-
OR7A10	-	Y	-
OR7A17	-	Y	-
OR7A5	-	Y	-
OR7C1	-	Y	-
OR7C2	-	Y	-
OR8B12	Y	-	-
OR8D2	Y	-	-
OR8I2	Y	-	-
OR9I1	-	Y	-
OR9Q1	-	Y	-
OR9Q2	Y	Y	-
ORMDL3	-	Y	-
OTOF	Y	-	-
PANX2	Y	-	-
PCDH19	Y	-	-
PCDH20	Y	-	-
PCDH7	-	Y	-
PCDH8	Y	-	Y
PCDHA10	Y	-	-
PCDHA11	Y	-	-
PCDHB15	Y	-	-
PCDHGA1	Y	-	-
PCDHGA3	Y	-	-
PCDHGA6	Y	-	-
PCDHGB1	Y	-	-
PCDHGB5	Y	-	-
PDGFRB	Y	-	-
PERLD1	-	Y	-
PIGN	Y	-	-
PKD1L1	Y	-	-
PKD1L2	Y	-	-
PKDREJ	Y	-	-
PLB1	Y	-	-
PLXDC1	-	Y	-
PMEPA1	-	Y	-
PODXL	Y	-	-
POPDC3	-	-	Y
PPAPDC1A	Y	-	-
PPAPDC1B	-	Y	-

PPAPDC3	Y	-	-
PPP1R3A	Y	-	-
PRPH2	-	Y	-
PRRG1	Y	-	-
PRRT1	Y	-	-
PTCHD3	-	-	Y
PTK7	-	Y	-
PTPLAD2	-	-	Y
PTPN1	-	Y	-
PTPRC	Y	-	-
PTPRR	-	Y	-
RCE1	Y	-	-
RGS9BP	-	Y	-
RHD	Y	-	-
RNF149	Y	-	-
ROBO2	-	-	Y
ROR1	Y	-	-
RTP1	Y	-	-
RYR1	Y	-	-
S100A10	-	Y	-
SCN3A	Y	-	-
SCNN1B	Y	-	-
SEMA4G	Y	-	-
SEMA5B	Y	-	-
SEMA6D	Y	-	-
SFXN3	Y	-	-
SGPP1	-	-	Y
SLAMF1	Y	-	-
SLAMF8	-	Y	-
SLC13A2	-	Y	-
SLC17A6	Y	-	-
SLC1A6	-	Y	-
SLC22A7	-	Y	-
SLC22A9	Y	-	-
SLC25A4	-	Y	-
SLC38A3	Y	-	-
SLC39A12	Y	-	-
SLC39A4	-	Y	-
SLC46A1	-	Y	-
SLC5A2	Y	-	-
SLC6A3	Y	-	-
SLC6A5	Y	-	-
SLC7A7	Y	-	-
SLC8A3	Y	-	-

SLC9A10	Y	-	-
SLC9A2	Y	-	-
SLCO2B1	Y	-	-
SLITRK1	-	-	Y
SORL1	Y	-	-
STAB1	Y	-	-
STARD3	-	Y	-
SUCNR1	-	Y	-
SUSD3	Y	-	-
SYNGR3	-	Y	-
SYT3	Y	-	-
TAS2R13	Y	-	-
TEK	Y	-	Y
TFRC	-	Y	-
TLCD1	-	Y	-
TLR3	-	-	Y
TM2D2	-	Y	-
TM4SF19	Y	-	-
TMED1	Y	-	-
TMEM123	Y	-	-
TMEM125	Y	-	-
TMEM132B	Y	-	-
TMEM14A	-	Y	-
TMEM14B	Y	-	-
TMEM161A	Y	-	-
TMEM179	-	Y	-
TMEM199	-	Y	-
TMEM206	Y	-	-
TMEM32	-	Y	-
TMEM37	Y	-	-
TMEM39A	Y	-	-
TMEM62	Y	-	-
TMEM63A	Y	-	-
TMEM82	Y	-	-
TMPRSS6	Y	-	-
TMTC4	Y	-	-
TNFRSF25	Y	-	-
TPCN2	-	Y	-
TPSG1	-	Y	-
TPTE	Y	-	-
TREM1	Y	Y	-
TREM2	-	Y	-
TREML1	Y	Y	-
TREML2	-	Y	-

TRPA1	-	Y	-
TRPC4	Y	-	-
TRPM7	Y	-	-
TSPAN2	-	Y	-
TSPAN4	Y	-	-
TXNDC15	Y	-	-
UNC50	-	Y	-
UTS2R	Y	-	-
VSIG8	-	Y	-
WBSCR28	Y	-	-
XKR7	Y	-	-
YIPF3	-	Y	-
YIPF6	-	Y	-
ZDHHC4	Y	-	-
ZDHHC5	-	Y	-

1 - Wood et al (2008)

2- Leary et al (2008)
